# How Home Delivery of Antiretroviral Drugs Ensured Uninterrupted HIV Treatment During COVID-19: Experiences From Indonesia, Laos, Nepal, and Nigeria

**DOI:** 10.9745/GHSP-D-21-00168

**Published:** 2021-12-31

**Authors:** Theresa Hoke, Moses Bateganya, Otoyo Toyo, Caroline Francis, Bhagawan Shrestha, Phayvieng Philakone, Satish Raj Pandey, Navindra Persaud, Michael M. Cassell, Rose Wilcher, Hally Mahler

**Affiliations:** a FHI 360, Durham, NC, USA.; b Achieving Health Nigeria Initiative, Akwa Ibom, Nigeria.; c FHI 360, Jakarta, Indonesia.; d FHI 360, Kathmandu, Nepal.; e FHI 360, Vientiane, Laos.; f FHI 360, Abuja, Nigeria.; g FHI 360, Washington, DC, USA.; h FHI 360, Hanoi, Vietnam.

## Abstract

**Introduction:**

Faced with the coronavirus disease (COVID-19) pandemic, governments worldwide instituted lockdowns to curtail virus spread. Health facility closures and travel restrictions disrupted access to antiretroviral (ARV) therapy for people living with HIV. This report describes how HIV programs in Indonesia, Laos, Nepal, and Nigeria supported treatment continuation by introducing home delivery of ARVs.

**Methods:**

Staff supporting the programs provided accounts of when and how decisions were taken to support ARV home delivery. They captured programmatic information about home delivery implementation using an intervention documentation tool. The 4 country experiences revealed lessons learned about factors favoring successful expansion of ARV home delivery.

**Results:**

Three of the countries relied on existing networks of community health workers for ARV delivery; the fourth country, Indonesia, relied on a private sector courier service. Across the 4 countries, between 19% and 51% of eligible clients were served by home delivery. The experiences showed that ARV home delivery is feasible and acceptable to health service providers, clients, and other stakeholders. Essential to success was rapid mobilization of stakeholders who led the design of the home delivery mechanisms and provided leadership support of the service innovations. Timely service adaptation was made possible by pre-existing differentiated models of care supportive of community-based ARV provision by outreach workers. Home delivery models prioritized protection of client confidentiality and prevention measures for COVID-19. Sustainability of the innovation depends on reinforcement of the commodity management infrastructure and investment in financing mechanisms.

**Conclusion:**

Home delivery of ARVs is a feasible client-centered approach to be included among the options for decentralized drug distribution. It serves as a measure for expanding access to care both when access to health services is disrupted and under routine circumstances.

## Abstract (precis)

During the COVID-19 pandemic, home delivery of antiretrovirals for HIV treatment proved to be a feasible approach for ensuring treatment continuation amid facility closures and travel restrictions. Antiretroviral home delivery is a model warranting further consideration as an additional option for decentralized drug delivery for HIV treatment.

## Teaser

**Key Messages:**

- Across diverse contexts, home delivery of antiretroviral (ARV) medications was a feasible and acceptable approach for ensuring access to HIV treatment when COVID-19-related lockdowns and travel restrictions imposed barriers to treatment.
- The ARV home delivery models were rapidly designed and successfully implemented to meet emergency needs brought on by the pandemic. Home delivery of ARVs requires further attention before it can be implemented at greater scale in response to the current pandemic and when health services face future shocks.

**Key Implications:**

- Governments and their partners are urged to seek financing mechanisms and reinforcement to commodity management systems needed to sustain this mode of decentralized service delivery.
- National HIV program leaders are encouraged to advocate for official, permanent policy changes supportive of home delivery of ARVs. Health policy makers should consider the relevance of home delivery to all chronic health needs requiring long-term delivery of medication to clients.

## INTRODUCTION

Governments worldwide rapidly instituted social distancing policies and lockdowns in 2020 to decrease the spread of severe acute respiratory syndrome coronavirus 2 (SARS-CoV-2), the virus that causes coronavirus disease (COVID-19).^1^^,^^2^ The restrictions disrupted routine health services, including initiation and continuation of antiretroviral therapy (ART) for people living with HIV (PLHIV).^3^^,^^4^ In some low- and middle-income countries (LMICs), health care workers have been redeployed to support the COVID-19 response, causing human resource shortages in routine services.^5^^,^^6^ Travel restrictions,^7^ clinic closures,^8^ and fears about the risk of infection at health facilities^9^ have discouraged health facility visits. The current COVID-19 pandemic and the possibility of future global health security threats restricting access to health facilities make it imperative to find alternative ways of delivering services to PLHIV in the interest of avoiding increased morbidity and mortality associated with treatment interruptions.^10^^,^^11^ The solutions are relevant not only to the continuity of HIV services but also to delivery of care for other health conditions requiring extended care, including TB, diabetes, and cardiovascular disease.^12^

Research evidence and programmatic experience in LMICs demonstrate the potential of differentiated service delivery (DSD) models for sustaining PLHIV on treatment.^13^ Endorsed by the World Health Organization in 2016,^14^ DSD describes client-centered service delivery approaches that adapt the timing, mode, and place of HIV services to the needs and preferences of a specific client population.^15^ DSD models include community-based provision of antiretroviral drugs (ARVs) to PLHIV established on ART through varied mechanisms, including client-led community adherence groups,^16^^,^^17^ provider-led adherence clubs,^18^ and community drug distribution points.^19^^–^^21^ Clients typically receive medication supplies lasting between 3 and 6 months through an approach known as multimonth dispensing (MMD).^22^ They are required to return to the health facility periodically for a clinical consultation (typically every 12 months) and viral load sample collection (Table). The COVID-19 pandemic has evoked calls for countries to accelerate the formulation of policies and implementation strategies supportive of both DSD^23^ and MMD^22^ that reduce the frequency of contact between health care workers and PLHIV, thereby reducing both bidirectional risks of SARS-CoV-2 transmission and burden on the health care system. Some approaches leverage the private sector through decentralized drug distribution through private pharmacies or automated dispensing.^24^

**TABLE.** Summary of Antiretroviral Drug Home Delivery Interventions in 4 Countries

	Nigeria	Indonesia	Nepal	Laos
Implementation period reported	May–Nov 2020	Apr–Nov 2020	Mar–Sep 2020	May–Sep 2020
Client groups^a^	General population/rural population	Key populations, their partners and children, and other priority populations	Female sex workers, MSM, transgender people, and migrants and their spouses	MSM/transgender people and their partners who are living with HIV
No. of facilities participating in home delivery	21	109	19	3
Recommended frequency of clinical consultation for established ART clients	Every 6 months	Every 6 months	Every 6 months	Every 6 months
Recommended frequency of viral load testing for established ART clients	Every 6 months	Every 12 months	Every 12 months	Every 6 months (for first year) and every 12 months for year 2 onward
Modification for viral load testing during the COVID-19 pandemic	Sample collection in the community	Transport of samples to private laboratories	Sample collection in the community	No change: facility-based collection required
Clients who received home delivery, n (% of eligible for home delivery)	4,138 (51)	4,948 (19)	2,836 (21)	126 (26)

Abbreviations: ART, antiretroviral therapy; MSM, men who have sex with men.

a Terms reflect client groups' preferred descriptions.

Research has shown the feasibility and effectiveness of a community-based strategy: ARV delivery within or close to clients' homes. Studies in Uganda,^25^^,^^26^ Kenya,^27^ and Tanzania^28^ have documented positive results associated with home delivery, indicated by improved adherence, treatment continuity, clinical outcomes, client satisfaction, and cost savings to the health system and clients.^29^ Despite the success of interventions tested on a small scale, they have not been incorporated widely into routine practice. National policies in many LMICs do not explicitly encourage ARV home delivery, and governments and their implementing partners have not invested in service delivery mechanisms to operationalize the practice. Drug supply shortages interrupt HIV programs' delivery of multimonth drug supplies; yet for operational costs to be affordable, deliveries should be limited to no more than 2–3 times per year.^18^ Other factors deterring expansion of home delivery of ARVs include intensive health system resource requirements needed for a high-functioning community health worker (CHW) program^30^ and persistent doubts about CHWs' ability to effectively deliver treatment for chronic conditions such as HIV/AIDS.^31^ Additionally, HIV programs are challenged with maintaining client confidentiality to minimize the risk of stigma and discrimination experienced by PLHIV when home deliveries are made.^32^

The COVID-19 pandemic has compelled HIV programs to overcome those and other barriers and to accelerate policy and programmatic change supporting ARV home delivery, not only during restrictions but as a client-centered model for populations inadequately served by other models. We present experiences from 4 LMIC settings where governments rapidly adjusted policies and service delivery mechanisms to provide ARVs to PLHIV in or near their homes. These 4 programs were selected based on the authors having direct access to programs where home delivery was rapidly introduced to adapt to COVID-19. The scenarios offer insights from experiences in geographically, culturally, and programmatically diverse settings. Program descriptions are followed by a discussion of lessons learned by technical advisors about the factors supporting and inhibiting ARV home delivery as a solution for sustaining clients on HIV treatment when access to facility-based services is compromised.

The COVID-19 pandemic has compelled HIV programs to accelerate policy and programmatic change supporting ARV home delivery as a client-centered model for populations inadequately served by other approaches.

## PROGRAM EXPERIENCES

With support from U.S. Agency for International Development (USAID) and the U.S. President's Emergency Plan for AIDS Relief (PEPFAR), FHI 360, an international nongovernmental organization supporting HIV programs in approximately 40 countries, provides technical assistance and implementation support to the featured HIV service delivery programs in Indonesia, Laos, Nepal, and Nigeria. To prepare this multicountry account, FHI 360 program staff serving as technical advisors retrospectively compiled programmatic information about HIV services and home delivery using an intervention documentation tool (Supplement). Staff provided background information on COVID-19-related lockdowns including when they were enforced and their influence on health service delivery and care-seeking practices. They also documented when and how policy change came about to support ARV home delivery.

**Figure:**
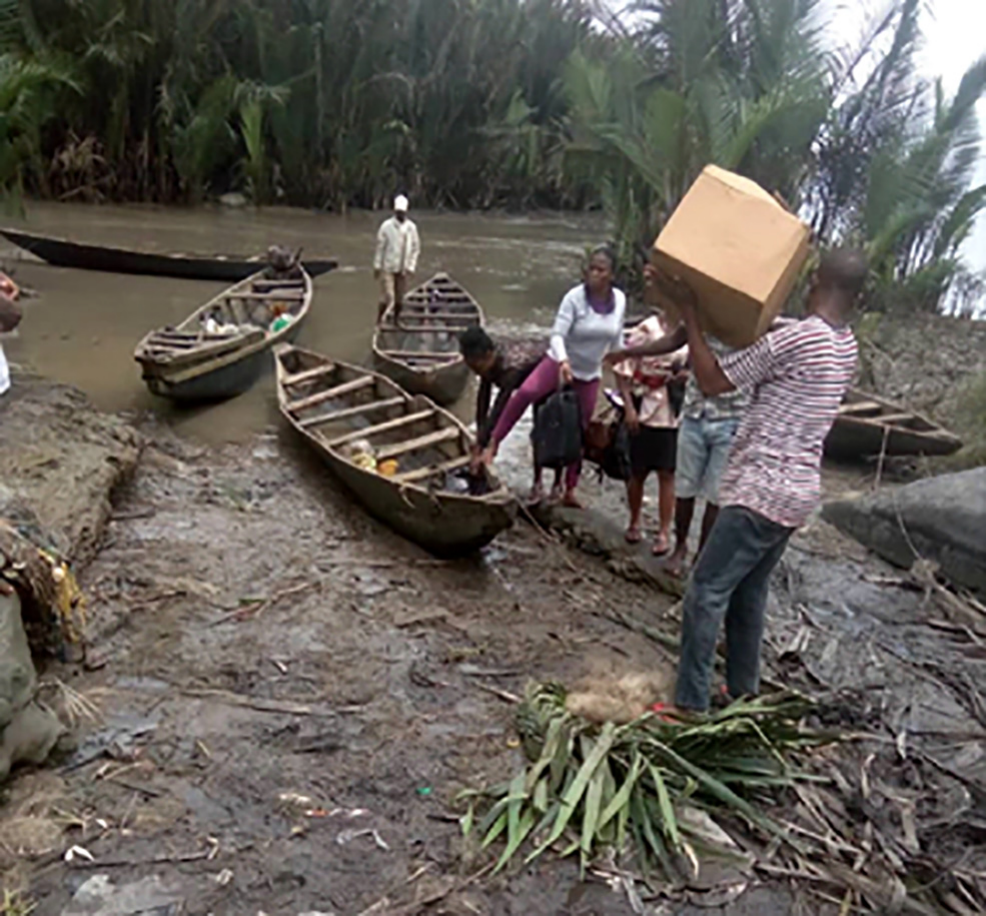
Discreet packaging of antiretroviral drugs in Jakarta, Indonesia. © 2020 Ade Sonyville and Arifin Fitrianto/LINKAGES/FHI 360 Indonesia

### Jakarta, Indonesia: Home Delivery Using Courier Services

The USAID- and PEPFAR-funded Linkages across the Continuum of HIV Services for Key Populations Affected by HIV (LINKAGES) project offers technical support to HIV programming in Jakarta province to ensure that key and priority populations living with HIV initiate and sustain treatment. In March 2020, the Jakarta Government responded to the COVID-19 pandemic by introducing large-scale social restrictions (known as “PSBB”) that limited domestic and international travel, imposed work-from-home policies on nonessential businesses, closed schools, constrained religious and cultural activities, and banned large social assembly. Public transportation was severely limited, and health facility staff were required to work alternating shifts, reducing facilities' ability to deliver care. The March 2020 provincial government circular specifically restricted face-to-face HIV community-based services, as mandated by the provincial government. By contrast, private sector services—particularly those that deliver essential goods—were exempted from large-scale social restrictions. Those transport companies put in place measures to protect their delivery staff, including PPE, sanitization protocols, and frequent rapid testing.

During the pandemic, private sector services were exceptionally well-placed to support ARV home delivery because they were considered essential services and were exempt from many large-scale social restrictions.

Recognizing this opportunity for private sector engagement, LINKAGES rapidly convened provincial health officials, clinical and community providers, and PLHIV representatives in virtual meetings to consider ARV home delivery. The Jakarta Provincial Health Office was initially reluctant to implement home delivery of ARVs due to concerns about maintaining confidentiality and financing the intervention. Decision makers were convinced after LINKAGES pledged to support technical, programmatic, and financial aspects of rollout and to work closely with provincial and district-level officials on monitoring this intervention. Once the Provincial Health Office introduced home-based ARV delivery into emergency regulations in March 2020, LINKAGES technical advisors joined provincial officials in establishing systems for ordering and transporting ARVs through Jak-Anter, a home-based ARV delivery system that uses ride-based apps and transport courier services. (*Anter* means “send” in Bahasa Indonesian.) Online training sessions were provided to socialize the idea of home-based delivery services with providers; ensure systematic application of standard operating procedures, particularly on packaging and ensuring informed consent and confidentiality; and establish recording and reporting procedures. LINKAGES also developed and rolled out demand-creation messaging for ART clients. Demand creation for Jak-Anter takes place via a variety of channels, including facility-based promotion by providers; service advertisements; face-to-face case management sessions with PLHIV; and social media platforms such as Facebook, Instagram, and TikTok.

Jak-Anter follows a multistep process to ensure the safe and secure delivery of ARVs from facilities to clients, beginning with contacting the client to confirm their address and desire for ARV home delivery. Providers in participating health facilities pack medication in containers concealing the contents. These are sent to the client through the Jak-Anter delivery system via a ride-based app or transport courier service such as Tiki, JNE, Grab, or Gojek. Facilities first pay the transportation fees and then are reimbursed by USAID/LINKAGES every week based on the number of clients served, as documented by clients with a phone message, photo, or WhatsApp message. Delivery firms use rigorous COVID-19 screening and safety protocols and provide PPE to protect drivers and clients, and they have committed resources for giving a one-time payout to any driver-partner who undergoes the government-mandated quarantine or tests positive for COVID-19.

Providers were initially reluctant to utilize Jak-Anter due to concerns about the timeliness of fee reimbursement and the added work of preparing, packaging, and arranging transport of medicine. Some PLHIV clients expressed worries about confidentiality and privacy, believing fellow community members could identify individuals with HIV infection through home-based drug delivery visits. Adoption increased over time, with the rise attributed to team-based provider incentives for patient retention, as well as improved service branding and promotion that emphasized protection of privacy. From March to December 2020, 4,948 unique PLHIV—or 19% of 26,203 PLHIV across 109 facilities—received home-based ARV delivery services through the LINKAGES' Jak-Anter system. Although retention specifically among Jak-Anter clients has not been analyzed, retention was more than 95% throughout 2020 among all ART clients in PEPFAR-supported sites in Jakarta. The Provincial Health Office incorporated home delivery into official policy and is working with LINKAGES technical advisors to scale the practice further through the Jak-Anter home-based ARV delivery system. The intervention is currently donor supported, with USAID/LINKAGES reimbursing health facility administrative costs and delivery costs. ARVs are the sole drug offered by the public sector health services through a home delivery system in Indonesia, with the Jakarta Provincial Health Office now exploring expansion of home-based delivery services for other medical treatment priorities.

### Laos: Home Delivery by Community-Based Supporters

In Laos, LINKAGES supports HIV services in 3 central hospitals in the capital city, Vientiane. People with positive tests were offered treatment through a variety of DSD models. Confronted with the COVID-19 pandemic, the Government of Laos enforced a full country lockdown from March 29 to April 19, 2020. Health facility access was permitted for emergencies only. PLHIV due for their ARVs could access health facilities through pre-appointment arrangement and only in the morning. Clients were required to carry a potentially stigmatizing letter from the hospital or their appointment card to show to the police while on the road; they also had to show this documentation to the guard before entering health facilities.

Recognizing how these restrictions reduced access to health services and threatened privacy, LINKAGES initiated home delivery of ARVs by collaborating closely with the Laos National Program for HIV, other implementing partners (IPs), UN agencies, and the Global Fund. Similar to the experience in Nepal, planning efforts involved rapid virtual engagement of governmental program officials, clinical and community providers, and PLHIV beneficiaries. Once the Ministry of Health authorized ART home delivery on an emergency basis in May 2020, ART sites established systems for drug ordering and transport. Health care providers offered outreach workers, known as community-based supporters (CBSs), on-the-job training to conduct the home-based delivery intervention; instruction covered confidentiality, consent, counseling, and safe delivery of ARVs, including COVID-19 prevention measures. ART sites issued CBSs a letter authorizing essential travel to present to police at checkpoints. After consulting records to identify stable ART clients who are eligible for MMD, the health care provider contacts the PLHIV using established communication lines via phone or social media to inform the client about the home delivery option, assess interest, and ask permission for the CBS to initiate the process. The CBS then contacts the client to offer home delivery or to agree on another place in the community to meet. For PLHIV who decline home delivery, the CBS assists with pre-appointment arrangement for a visit to health facilities. Initially, home delivery packages had drugs lasting 3–4 months for stable PLHIV; this increased to 4–5 months by the end of June 2020. In conducting home/community delivery of ARVs, CBSs are expected to follow the same protocol for privacy and confidentiality that is used for home visits to PLHIV for the provision of adherence and retention support. Regarding protections from COVID-19, the outreach workers travel to communities on their motorbikes, and they are provided face masks and hand sanitizer.

Once the Ministry of Health in Laos authorized ART home delivery on an emergency basis in May 2020, ART sites established systems for drug ordering and transport.

Between May and September 2020, a total of 126 PLHIV had received home delivery, accounting for 26% of 480 individuals who were due for refill. Those who declined this service were more apt to be living with friends or parents or on a military base; others declined because they had established a close rapport with the facility-based health care providers and wanted to maintain that contact. Although home delivery is just one factor influencing HIV program outcomes, viral load suppression remained above 95% throughout 2020 in the PEPFAR-supported sites. With the ease in restrictions in Laos, home delivery of ARVs continues to be an attractive option for some clients who live far from the hospital or who struggle financially to travel to the hospital. The intervention depends on donor support, with transportation and lunch allowances for CBSs paid by either LINKAGES or the Global Fund ($5 to $8.50 per client served).

### Nepal: Home Delivery by CHWs

In Nepal, LINKAGES collaborates with the national government, 21 IPs, and national networks of key populations and PLHIV to deliver HIV services in 19 of the country's 77 districts. The COVID-19 nationwide lockdown raised an urgent need for service delivery adaptations to support clients in continuing ART. All LINKAGES-supported Nepal city clinics were closed during the lockdown period (March–July 2020); local orders dictated that clinics remain closed, and movement continued to be restricted in districts with more than 200 active COVID-19 cases. As of November 2020, only 3 of 19 LINKAGES-supported clinics were open, and ART sites that were open operated with limited opening hours and capacity.

The COVID-19 nationwide lockdown in Nepal raised an urgent need for service delivery adaptations to support clients in continuing ART.

Nepal's 2020 National HIV Testing and Treatment Guidelines support community-based ART and ARV dispensing sites as a DSD model, without a specific policy supporting home-based ARV delivery. To adapt to COVID-19-related restrictions, LINKAGES rapidly engaged stakeholders to plan and initiate home delivery. Collaborators included representatives of the National Center for AIDS and STD Control (NCASC), IP organizations, clinic- and community-based ART service providers, and PLHIV networks. CBSs and peer navigators affiliated with IPs, are the frontline implementers of the home delivery intervention in 19 project districts. LINKAGES Nepal IPs collaborate with 29 ART sites in preparing lists of PLHIV who are due for a refill. IP staff members contact clients to inform them that their treatment is available at the ART site or community-level facility. PLHIV have the option of picking up ARVs from designated ART sites or city clinics or they can opt to have the outreach worker deliver ARVs to their home or to a chosen location in the community. To date, there has been no official change in national policy authorizing ARV home delivery, although it has become an acceptable component of existing community-based services. Between March 1 and September 30, 2020, LINKAGES-supported partners in Nepal provided ARV drugs to 2,836 PLHIV in or near their homes, accounting for 21% of all eligible PLHIV within the supported sites. One factor limiting use of home delivery was that PEPFAR services were transitioning to the use of tenofovir, lamivudine, and dolutegravir, a treatment requiring patients to visit health facilities. Although no data are available showing how home delivery affected retention, Nepal's overall 12-month retention in 2020 was 96.7%.

**Figure:**
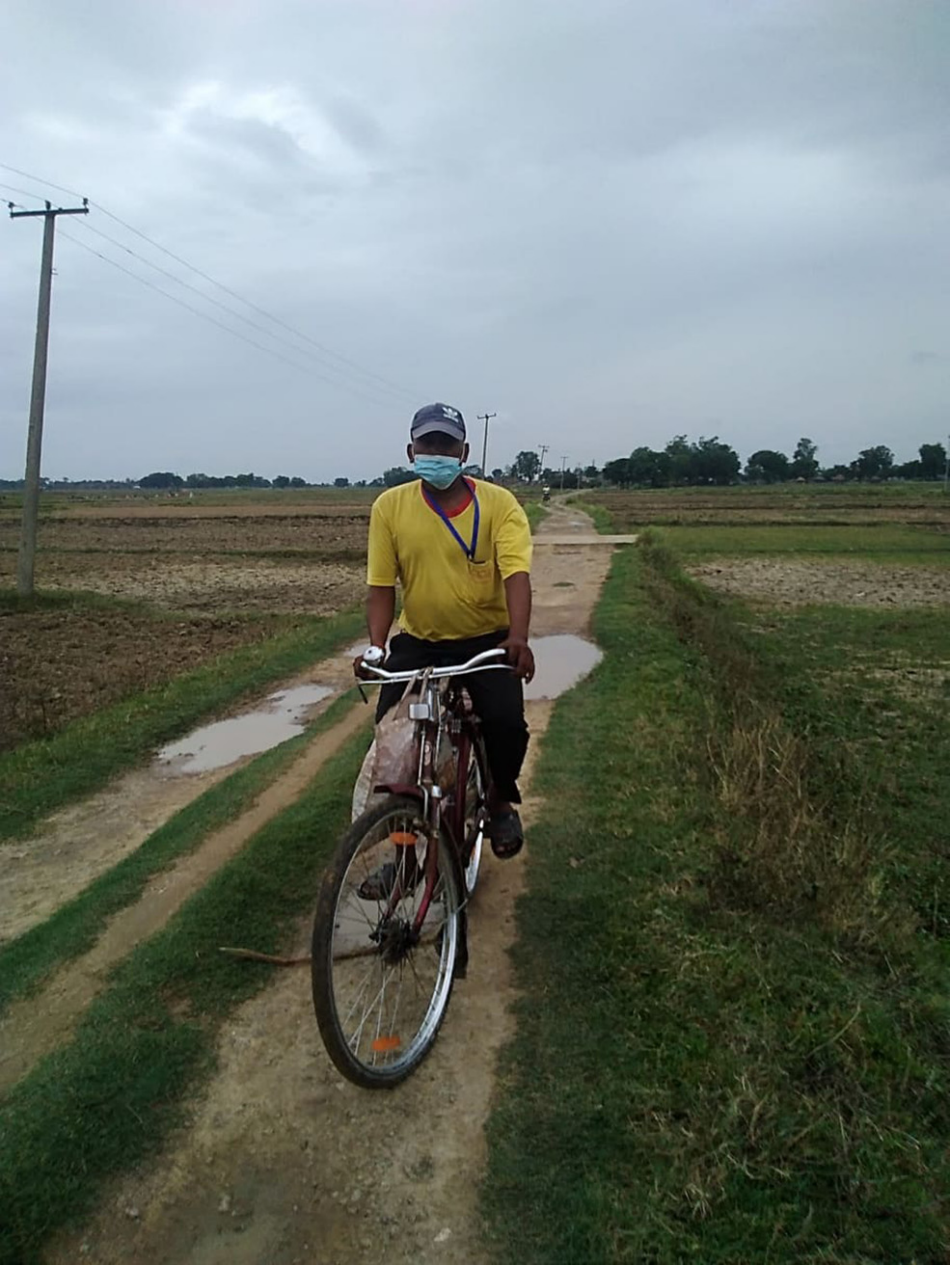
Outreach worker on the way to delivering antiretroviral drugs to clients at their homes in Nepal. © 2020 NAMUNA/LINKAGES/FHI 360

### Akwa Ibom State, Nigeria: Home Delivery by CHWs

About 6 months before COVID-19 was first detected in Nigeria, home delivery of ARVs was first introduced in a large primary health facility in Akwa Ibom state—where the USAID-funded Strengthening Integrated Delivery of HIV/AIDS Services (SIDHAS) project supports the government in providing HIV treatment services in 102 health facilities. Noting in March 2019 that only 73% of those who had started ART 12 months prior were still receiving care, program staff considered DSD approaches to make treatment more patient centered and accessible. Community ART refill groups were not regarded as a good option because of stigma and the wide geographic dispersion of clients' households in Mbo, a local government area (LGA) where the facility is located. Supported by Nigeria's National Policy on Task Shifting and Task Sharing for Essential Health Care Services, health center staff together with SIDHAS technical advisors decided to initiate home delivery of ARVs in August 2019. The service was offered to clients established on ART, defined as those on ART for at least 3 months, who had no opportunistic infections and were free of adverse drug reactions.

**Figure:**
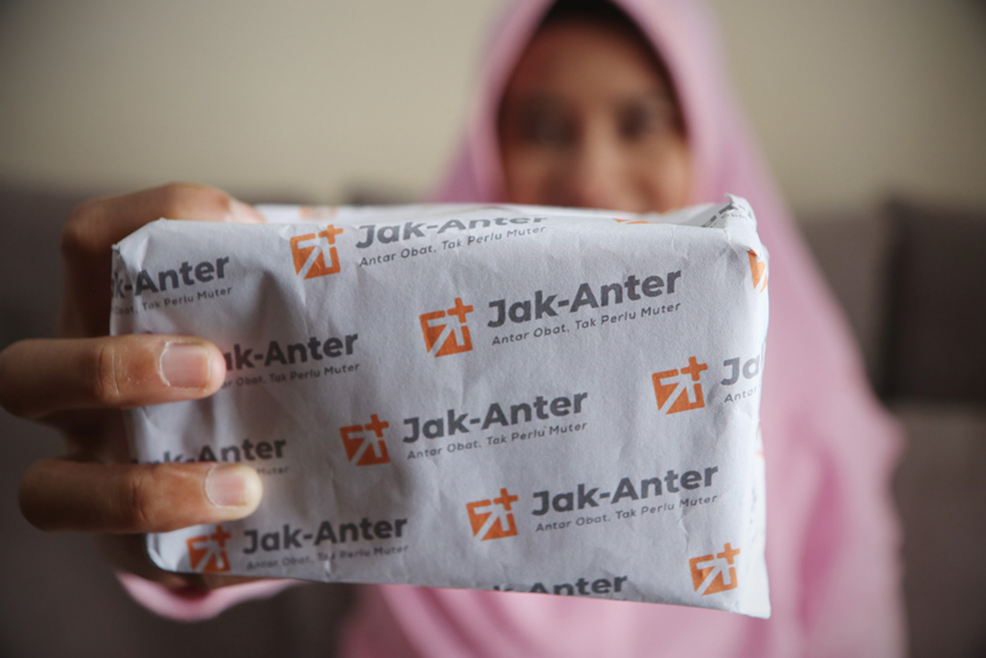
A community pharmacist boards at the jetty with supplies for clients along remote creeks in Akwa Ibom State, Nigeria. © 2020 Otoyo Toyo/AHNI

Home delivery of ARVs took on greater importance when the COVID-19 lockdown was enforced in Akwa Ibom state in mid-March 2020. At the height of restrictions (April–May 2020), vehicular movement was prohibited, although health care workers were permitted to travel with an Essential Duty Pass. Limited supplies of face masks and their prohibitive cost when they were available further deterred clients from accessing health facilities. Some clients in the 21 LGAs served by SIDHAS refrained from going to health facilities due to the fear of contracting the virus: 9,303 of 65,288 clients (14%) missed HIV clinic appointments in the 2 months preceding the lockdown, a time when media reported COVID-19 infections in Nigeria. Home delivery was extended from 21 to 80 health facilities to ensure uninterrupted refills. This extension required engaging with the clients, state and LGA officials, health facility staff, and civil service organizations such as the Network of People Living With HIV/AIDS in Nigeria (NEPWHAN); training health care providers and outreach workers on the new model; and activating toll-free numbers to provide ongoing support to the clients.

Home delivery of ARVs took on greater importance when the COVID-19 lockdown was enforced in Akwa Ibom State in mid-March 2020.

In this model, health facilities use client data to generate weekly lists of clients eligible for home refills; clients are clustered by location, and case managers are assigned to provide the service. Community health extension workers, case managers, and patent medicine vendors have been trained by pharmacists to collect prepacked ART from respective facilities and to deliver it to clients at home or an alternative pick-up location proposed by the client, such as churches, schools, shopping malls, or market stalls. Program staff travel by boat, canoe, motorcycle, tricycle (“keke”), and on foot to reach the households. The program provides outreach workers with personal protective equipment (PPE) (face masks and sanitizers) and screens them for COVID-19 symptoms. When health facilities contact clients by phone to schedule a home visit, they assess knowledge about COVID-19 and presence of COVID-19 symptoms; clients with symptoms are referred to the state COVID-19 response team for testing. SIDHAS helps to cover CHWs' transportation costs using funds designated for tracking clients through home visits.

Between May and November 2020, 4,138 of the 8,136 (51%) clients on ART in Mbo LGA were receiving prepacked ARVs by home delivery, with overall client retention at 99%. Reflecting on their experiences with patients who chose not to use home delivery, program implementers posited that this could be due to concerns about confidentiality, privacy, and stigma or to a preference for other DSD options that are convenient for them, such as fast track clinics or refill clubs.

## LESSONS LEARNED ON ACCELERATING INTRODUCTION AND EXPANSION OF HOME DELIVERY OF ARVS

The 4 country programs spanning diverse LMIC contexts demonstrate the feasibility of home delivery as a routine DSD approach for HIV treatment in LMICs. The following are key lessons learned from those experiences from the perspective of technical advisors who supported the programs.

The 4 country programs spanning diverse LMIC contexts demonstrate the feasibility of home delivery as a routine DSD approach for HIV treatment in LMICs.

### Rapidly Engage Stakeholders

Rapid stakeholder engagement to design and endorse service innovations was instrumental to the success of these 4 HIV programs in adapting successfully to the COVID-19 pandemic. With the World Health Organization's March 2020 pandemic declaration and the national and local restrictions that quickly followed, HIV program partners recognized the urgency of service delivery adjustments to ensure continuity of care for ART clients. Each of the programs engaged virtually with stakeholders, including governmental health program officials, clinical and community providers, and PLHIV beneficiaries. Policy adjustments required intensive, sustained dialogue advocating for the safeguarding benefits of home delivery for both PLHIV beneficiaries and providers. Review of client data reflecting missed appointments and travel distances helped to make the case that home delivery would greatly support clients and improve retention in HIV care.

Involving PLHIV and their advocates in intervention design and launch of service delivery innovations was an essential form of stakeholder engagement. Their input allowed interventions to be tailored to beneficiaries' varied needs and preferences. In Laos, for example, program implementers were advised home delivery would be more effective if it is conducted by PLHIV support groups, community organizations, or the staff of services like the Youth Clinic. Similarly, in Nepal, the intervention involved mobilizing and building the capacity of PLHIV CBSs and peer navigators to conduct home delivery, capitalizing on their thorough knowledge of the community.

Institutionalization of home delivery as a standard of care requires supportive policies. None of the 4 countries have policies explicitly supporting ART home delivery. Jakarta is in the process of putting home-based care into policy at local levels, and the circular letter instituted in March 2020 provided the impetus for broader policy discussions. Facing the COVID-19 emergency, government officials recognized that programs needed stop-gap solutions and reacted quickly by approving home delivery on an emergency basis. For the service innovation to be implemented permanently, decision makers will need to reconvene to effect official policy change. Decision making should be guided by examining initial home delivery experiences and considering practical plans for implementing home delivery at scale. Engaging in advocacy for client-centered approaches, HIV programs could be aided by joining forces with representatives of other health programs that could more effectively serve clients requiring long-term drug therapy through home delivery of treatment.^33^ Extending home delivery to include the provision of medications for other chronic conditions would benefit clients while also potentially increasing efficiency for the health system.

### Invest in DSD Models

Differentiated models of care allow ART programs to adapt to contextual shocks and to avoid service disruptions. Governments and their IPs were able to adapt to the COVID-19 emergency through ARV home delivery due to pre-existing policies and practices supporting DSD. MMD, the practice of providing virologically suppressed clients with ARV refills every 3 or 6 months, contributed to the feasibility of ARV home delivery; visiting clients more frequently would have been more expensive and logistically difficult. Although support for MMD has grown markedly in PEPFAR-supported countries in recent years, the supply chain infrastructure requires reinforcement to make this the universal service delivery norm.^34^ A growing base of successful experience with community-based provision of ARVs by outreach workers without clinical credentials laid the foundation for rapid approval of home delivery in the face of the COVID-19 crisis.^35^ Continued investment in DSD models is warranted to alleviate the burden on already overstrained health facilities and to prepare for future shocks threatening service continuity. Additional evidence is needed to refine the models, test alternatives, and assess their cost-effectiveness.^13^ Attention should also be focused on the professionalization of the nonclinically credentialed outreach workers responsible for community-based ARV delivery, to the extent that it is a factor impeding formal policies supporting their role. Notably, peer educators, peer navigators, and other community-based cadres are increasingly recognized as an essential part of a country's health workforce. In 3 of the 4 case examples provided in this article, home delivery of ART was provided by community-based health workers. National policymakers should acknowledge the essential leadership role that communities play in the delivery of health services through increased domestic financing of community-led services, including home delivery of ART.

Pre-existing policies and practices supporting DSD helped governments and their IPs to adapt to the COVID-19 emergency with ARV home delivery.

### Protect Outreach Workers and Clients

Protections for outreach workers and clients are essential features of home delivery approaches. Foremost, measures were introduced to minimize the risk of SARS-CoV-2 transmission between individuals delivering ART (outreach workers/rideshare employees) and clients. Programs developed detailed protocols to screen CHWs for COVID-19 symptoms, to refer suspected cases to the public health system for testing, to quarantine individuals who were potentially infected, and to link people with positive test results to care. HIV programs also developed and disseminated materials with educational messages on preventive measures such as physical distancing and hand washing. Finally, workers responsible for home delivery received PPE such as face masks, gloves, and hand sanitizer.

Another fundamental protective measure was the emphasis on ensuring client confidentiality. Across countries, a factor presumed to inhibit acceptance of home delivery among some clients was fear of inadvertent disclosure of HIV status that could place the client at risk of stigma or violence. Programs introduced measures for privately contacting the clients to obtain consent and to assess and minimize the risk of violence or other harm that may be associated with home delivery of ART. These built on pre-existing processes for obtaining consent for home visits for adherence support sought from all clients as they initiate ART. Programs packed medicines discreetly so the contents could not be identified. For clients with remaining concerns about undesired disclosure of their HIV status to co-residents, programs pivoted to offer delivery alternatives. For example, outreach workers arranged to meet PLHIV clients somewhere outside their homes, such as at the bus station, the village entrance, or the park.

### Ensure Intervention Sustainability

For home delivery to continue beyond emergency situations, like the current COVID-19 pandemic, mechanisms are needed to ensure intervention sustainability. A reliable ARV supply chain is essential to supporting the sustainability of home delivery. At the initial peak of the COVID-19 pandemic, disruptions in international air shipments and national transport threatened the supply chain. When programs were initially challenged in implementing MMD due to strained drug supplies, home delivery allowed frequent dispensing while reducing client and provider COVID-19 exposure risk that would occur in busy health facilities. For home delivery to be efficient, however, programs must have ARV supplies sufficient to allow MMD. High-level stakeholder engagement in some of the countries resulted in some improvements in commodity management but concerns about potential ARV shortages persist. For home delivery to be offered permanently at a broader scale, forecasting must be more streamlined based on client projections and accurate consumption data.

For home delivery to be offered on a permanent basis at a broader scale, forecasting must be more streamlined based on client projections and accurate consumption data.

The sustainability of home delivery also depends on HIV programs devising new health financing measures to support the intervention. Home delivery was necessary for many clients to prevent treatment interruptions at the peak of COVID-19. The model will still be needed as countries experience additional waves of the pandemic. Ongoing funding will be required to support home delivery for specific client groups, and costs associated with home delivery need to be systematically assessed to inform sustainability discussions.

The 4 country scenarios were possible through the infusion of supplementary resources offered by donor-funded projects already supporting HIV services. Even with external support, resources are insufficient to extend home delivery in time and space. In Nigeria, for example, serving the increasing numbers of patients opting for home-based refills has become too costly for its HIV program to sustain. Increased support for DSD models that include community participation and task-sharing will build the infrastructure necessary to sustain home delivery.^36^ Just as community-led services have been critical to an effective HIV response in LMIC,^36^ these services have proven to be essential to HIV programs' resilience during the COVID-19 pandemic. Increased investment in community-led services will help build the infrastructure necessary to sustain home delivery. Programs can justify investment in all the supports required for home delivery by producing programmatic data and completing cost-effectiveness modeling showing that it is a valuable addition to the ARV refill options, contributing to increased retention of clients in care, a persistent challenge to national HIV programs.^37^^–^^39^

### Limitations

The featured home delivery interventions were introduced independently and at different times in each country in response to the pandemic. Under these emergency circumstances, a valid comparator was not put in place, thereby limiting conclusions that can be drawn about the public health impact of the intervention. Similarly, mechanisms were not introduced to track individual clinical outcomes or to capture the resources required to prepare and implement the home delivery interventions, thereby precluding cost-effectiveness analyses. The case studies were primarily based on program implementers' accounts and did not include primary data collection with providers or clients; the accounts may therefore be favorably biased. Finally, the featured programs were well financed due to donor support. The Akwa Ibom program was part of a focused PEPFAR initiative known as a surge, resulting in high numbers of clients initiating ART. The documented experiences are likely not transferable to programs with dire resource constraints.

## CONCLUSION

The home delivery model is a client-centered, community ART management program that improves the quality of life for PLHIV by providing a convenient means of uninterrupted access to ART. Experiences in the 4 countries suggest that inclusive engagement of all stakeholders, swift adoption of supportive policies, and service delivery models that are responsive to client preferences help to create feasible, acceptable home delivery interventions. Community-based and key-population-led service providers were poised to advance the differentiated service delivery approaches that COVID-19 necessitated, including home delivery of ART. Continuing to invest in communities and give them the legal and political legitimacy to operate in a way that reflects how important they are to health system functioning could help bolster health systems' flexibility and resilience in the face of future pandemics. Providing home delivery of ARV medications is a promising option to ensure safe and sustained access to lifesaving HIV treatment among PLHIV in places where COVID-19-related lockdowns, quarantines, and physical distancing restrictions may impose substantial barriers to treatment retention. The documented programs show that home delivery could complement other out-of-facility models that could be rapidly activated to ensure treatment continuity in the face of future pandemics or localized disasters. Implementation experiences illustrate how programs can reduce access gaps by responding to client needs and preferences and adapting to external circumstances.

Rigorous evaluation is still needed to examine how home delivery complements and compares to other DSD models in terms of its impact on treatment continuity and clinical outcomes like viral suppression. Such research should explore the circumstances under which home delivery fills needs not met by other DSD models, an issue that could be informed through primary data collection with clients, providers, services managers, and community stakeholders. Also needed is a systematic assessment of the financial, human, logistical, and material resources required to sustain home delivery in different contexts. Such evidence could be used to guide thoughtful design and implementation of well-conceived responses to future pandemics and other shocks limiting access to health services. In the context of the current COVID-19 crisis, we propose that governments and donors support rapid scale-up of the home delivery DSD model to ensure that PLHIV can be retained on lifesaving treatment during these extraordinary times. For maximum resilience when confronted with other threats to HIV service continuity, HIV programs are encouraged to explore options for sustaining home delivery of ARVs as well as other services such as HIV testing services, pre-exposure prophylaxis, and family planning.

## Supplementary Material

21-00168-Hoke-Supplement.pdf

## Notes

**Peer Reviewed**

**First published online:** October 12, 2021.
