# Supplementary material for: How Home Delivery of Antiretroviral Drugs Ensured Uninterrupted HIV Treatment During COVID-19: Experiences From Indonesia, Laos, Nepal, and Nigeria
Source: Glob Health Sci Pract. 2021 Dec 31;9(4):978–89. doi: 10.9745/GHSP-D-21-00168 (PMC8691873; doi:10.9745/GHSP-D-21-00168)
Supplement: 21-00168-Hoke-Supplement.pdf [file 21-00168-Hoke-Supplement.pdf]

Supplement to: Hoke T, Bateganya M, Toyo O, et al. How home delivery of antiretroviral drugs ensured uninterrupted HIV treatment during COVID-19: experiences from Laos, Indonesia, Nepal, and Nigeria. *Glob Health Sci Pract*. 2021;9(4). https://doi.org/10.9745/GHSP-D-21-00168

Supplement

Profile reflecting home-based delivery of ARVs in the context of COVID-19

Context:

1	Cumulative number of reported COVID-19 cases to date	(provide date)
2	Describe current restrictions on the operations of health facilities.	
3	During the period when restrictions were most severe, what restrictions were placed on facility-based health services? During what time period were these restrictions in place?	
4	Describe any restrictions impacting travel or movement within towns/cities in response to COVID-19. Describe how these restrictions have changed over time.	
5	Describe policies or service delivery guidelines in place January 1, 2020 (pre-COVID) related to home-based delivery of ARVs.	
6	Describe any changes in policies (#5) or service delivery guidelines that have occurred in response to COVID- 19.	
7	Briefly describe how the change in policy or guidelines (#6) came about. Who advocated for the change? Who made the decision to change the policy? Briefly explain the process by which the change came about.	
8	Explain any ways that ART clients or their advocates participated in the design or launch of the home-based services.	
9	Summarize how home-based delivery of ARVs is being conducted now.<br>• What cadre makes the home deliveries?<br>• With what frequency?<br>• What logistical support does the cadre receive to do home deliveries, and from whom?<br>Provide any key details to describe how services operate.	

10	How many clients in total have benefited from home-based delivery of ARVs since this service mode began?	
11	Describe how the monthly number of clients benefiting from home-based delivery of ARVs has changed from the pre-COVID period to now.	
12	What components of the system supporting home-based delivery of ARVs are working most successfully? What factors contribute to this success?	
13	What components of the system supporting home-based delivery of ARVs have been more problematic or challenging?	
14	What suggestions do you have about replicating home-based delivery of ARVs in other contexts?	
